# 

**Published:** 1879-04

**Authors:** 


					﻿APRIL, 1879.
TWENTY-SIXTH YEAR—No 304.
HALL’S
Journal of Health.
PUBLISHED AT 141 EIGHTH STREET,
(Near Broadway),	•	TSJ'E'VT" “TORK.
E. H. GIBBS, A.M., M.D., Editor.
AGENTS FOR THE TRADE:
AMERICAN NEWS COMPANY AND NEW YORK NEWS COMPANY
Terms, $1.50 a Year, or $1.00 for Eight j'looBis. Postage paid.
SINGLE NUMBER, 15 ¿jx>9.
Couch & Johnston, Printers, 11 Frankfort Street, New York.
				

